# Abnormal white matter integrity in rapists as indicated by diffusion tensor imaging

**DOI:** 10.1186/s12868-016-0278-3

**Published:** 2016-07-07

**Authors:** Chiao-Yun Chen, Adrian Raine, Kun-Hsien Chou, I-Yun Chen, Daisy Hung, Ching-Po Lin

**Affiliations:** Department and Graduate Institute of Criminology, National Chung Cheng University, Chiayi, 621 Taiwan; Department of Criminology, Psychiatry, and Psychology, University of Pennsylvania, Philadelphia, PA USA; Brain Research Center, National Yang-Ming University, Taipei, 112 Taiwan; Institute of Neuroscience, National Yang-Ming University, Taipei, 112 Taiwan; Institute of Cognitive Neuroscience, National Central University, Jhongli, Taiwan

**Keywords:** Rapist, Reward, Moral judgment, Diffusion tensor imaging, White matter

## Abstract

**Background:**

Recent research has documented structural brain abnormalities in various criminal offenders. However, there have been few brain imaging studies of sex offenders, and none on white matter integrity. The current study tested the hypothesis that rapists, when compared to matched controls, would show abnormal cortical and subcortical white matter integrity.

**Results:**

Rapists showed significantly increased fractional anisotropy in the internal capsul e in the thalamus, caudate, and globus pallidus, and also in white matter tracts near the angular gyrus, posterior cingulate, frontal pole, lateral occipital cortex, and genu compared to controls matched for age, gender, and educational status. Reduced fractional anisotropy was observed in rapists in the posterior cingulum and in the inferior fronto-occipital fasciculus.

**Conclusions:**

To our knowledge, this is the first study indicating white matter abnormalities in rapists. Findings indicate abnormalities in white matter connectivity in brain regions involved in reward/motivation and moral judgment, which may predispose rapists to be both over-responsive to sexual reward stimuli and also to make inappropriate moral decisions.

## Background

In 2011, The National Center for Missing and Exploited Children estimated that there were over 747,000 registered sex offenders in the United States. As the rate of sex crimes and number of sexual offenders continue to grow, much effort has been devoted to understanding the nature of these crimes, as well as the characteristics of the offenders. Some have proposed that heterosexual rapists may be over-responsive to sexual stimuli [1]. Others have hypothesized an association between the construct of moral judgment and sexual offenses [e.g., 2, 3]. In recent years, such investigations have benefited from advances in neuroimaging technologies, which contribute to better understanding of the brain areas that may be involved with the commission of sexual crimes. For example, Schiffer et al. [4] found that the thalamus, globus pallidus and striatum, which correspond to key brain areas involved in sexual arousal and behavior, showed significant activation in pedophiles, but not in controls. Research on moral decision-making has also documented abnormalities in the neural circuitry involved in moral decision-making in antisocial individuals, specifically, the medial polar prefrontal cortex, the angular gyrus, the amygdala, and the posterior cingulate [5, 6].

Despite the increased utilization of imaging technology, few studies have examined the integrity of white matter, as opposed to functional or structural imaging. Though several studies have documented reduced fractional anisotropy (FA) in association with increased aggression in schizophrenics [7–9], we are currently not aware of any study that has investigated FA in sex offenders. The current study examined white matter integrity in sex offenders using diffusion tensor imaging (DTI) to assess FA in the brains of 15 adult males who had raped one or more adult females, and 15 matched controls. Based on the aforementioned research findings, we hypothesized that abnormality would be observed in some of the brain structures involved in sexual arousal and behavior among rapists, including those constituting the basal ganglia. In addition, we hypothesized that sex offenders would also show abnormality in the white matter circuitry connected with the medial polar prefrontal cortex, the angular gyrus, the amygdala, and the posterior cingulate.

## Methods

### Subjects

Participants consisted of 15 male sex offenders and 15 controls, matched for age and gender. There were no differences between the sex offender (mean age 33.1 ± 6.5 years) and control (mean age 33.0 ± 6.8 years) groups in age (t_.05_(14) = 0.04, *p* = 0.968).

Sex offenders were recruited from Taipei Prison in Taiwan. In an effort to delineate a relatively homogenous group of sex offenders, offenders had to be male adults who had raped an adult female stranger. They were serving sentences ranging from 5 to 10 years. Exclusion criteria for this study were a history of psychiatric illness, neurological illness, prior and current psychiatric treatment, and commission of rape while under the influence of alcohol or drugs. The non-offender control subjects were recruited from the Center of Health Examination in Taipei Veterans General Hospital, Taiwan. None of the control participants had any history of psychiatric or neurological illness, previous or current psychiatric treatment, or history of drug or alcohol abuse. All participants provided written informed consent before participating in the study, which was approved by the Ethics Committee of Taipei Prison, Taiwan and the Institutional Review Board of National Yang Ming University (ref: 970003).

### Data acquisition

The magnetic resonance imaging (MRI) scans were performed on a 1.5 T MR system (Excite II; GE Medical Systems, Milwaukee, Wis., USA) equipped with an 8-channel head coil in Taipei Veterans General Hospital. All subjects underwent the same imaging protocol using T1-weighted (T1 W) and diffusion-tensor imaging. Images were acquired parallel to the anterior–posterior commissure line. High resolution T1 W structural images covering the entire brain were acquired using three dimensional fluid-attenuated inversion-recovery, fast spoiled gradient recalled echo (FLAIR-FSPGR) sequence with following parameters: repetition time (TR) = 8.548 ms, echo time (TE) = 1.836 ms, inversion time (TI) = 400 ms, flip angle = 15°, field of view = 260 × 260 mm^2^, matrix size = 256 × 256, number of slice = 124 and slice thickness = 1.5 mm.

DTI scans were acquired using a single-shot spin-echo echo-planar imaging (EPI) sequence with diffusion sensitization gradients applied in thirteen non-collinear directions at a b-value of 900 s/mm^2^. Additional null (b = 0 s/mm^2^) images were acquired as reference images for signal attenuation measurement. The following parameters were used: TR = 17,000 ms, TE = 68.9 ms, number of excitations (NEX) = 6, number of slices = 70 in the axial orientation for whole brain coverage, slice thickness = 2.2 mm without slice spacing, FOV = 260 × 260 mm^2^, and matrix size = 128 × 128. Total scanning time for each subject was approximately 32 min.

### Image processing

DTI preprocessing, including eddy current correction and brain tissue extraction using the Brain Extraction Tool (BET v2.1) [10], was performed with FSL v4.1.7 (Functional Magnetic Resonance Imaging of the Brain Software Library; http://www.fmrib.ox.au.uk/fsl). The eddy current correction involved registering the diffusion-weighted images to the null image through affine transformations not only to minimize the image distortions due to the eddy currents induced by the fast-switching gradient coils, but also to reduce the simple head motion. The Brain Extraction Tool was applied to remove the non-brain tissue and background noise from the images. Voxel-wise calculation of fractional anisotropy images were derived based on Basser’s model [11] using in-house software.

### Statistical analysis

The Tract-Based Spatial Statistics (TBSS) tool in FSL was used to calculate differences in FA values between the sex offender group and controls. TBSS is described in greater detail elsewhere [12]. In general, the strengths of voxel-based-analyses (VBA) are that they are fully automated, and do not require pre-specifying features or region-of-interest. However, errors and concerns may be raised due to alignment inaccuracies and the lack of a principled way for choosing a smoothing extent. In Tract-Based Spatial Statistics (TBSS), the idea of projection of FA data onto a WM skeleton is to avoid the partial volume effect (PVE) and gain statistical power since this approach does not require data smoothing. Meanwhile, TBSS keeps the features of a fully automated method [13].

Like all methods, this approach also has some drawbacks that should be recognized when considering analysis outcomes. TBSS discards the orientation information captured in the diffusion data since it only makes use of FA maps. The extent of anatomical inaccuracies, which is inherent in the FA skeleton projection, can potentially introduce biases in the data outcomes. Additionally, TBSS is known to be purely FA-based and it has been previously reported that adjacent WM tracts can be not necessarily separable based only on their FA [14, 15].

For the analysis, briefly, a cross-subject mean FA skeleton, which was previously aligned to the MNI-152 template, was created to represent the centers of all fiber tracts common to the entire group. All other FA maps were transformed to the MNI-152 space by concatenating the FNIRT nonlinear transformation to the representative FA image with the transformation to the MNI-152 template. Each subject’s aligned FA map was projected onto the mean FA skeleton by filling the skeleton with FA values from the nearest relevant tract center. This ensured that each subject’s skeleton was in the group space, yet represented the center of that subject’s own unique fiber tracts. The skeletonized FA data were fed into the following voxel-wise cross-subjects statistics which were based on non-parametric permutation testing (Randomise v2.1, a part of the FSL tool; http://www.fmrib.ox.ac.uk/fsl/randomise/index.html).

Analysis of between-group difference in FA values were conducted in a non-parametric analysis of covariance design [16] using a cluster-extent threshold to account for the multiple comparison (corrected-p < 0.05). Age was entered into the analysis as a potential confound to ensure that any observed difference in FA between the two groups was independent of age-related changes. Each possible contrast (normal control > sex offenders and sex offenders > normal controls) was tested with 5000 random permutations. Effect sizes were calculated using Cohen’s d [17]. The most probable anatomic localization of each cluster was determined by means of the FSL atlas tool (http://www.fmrib.ox.ac.uk/fsl/fslview/atlas-descriptions.html). All reported brain images were acquired using the “tbss_fill” script from the FSL package.

## Results

### Direct group comparisons

**Fig. 1:**
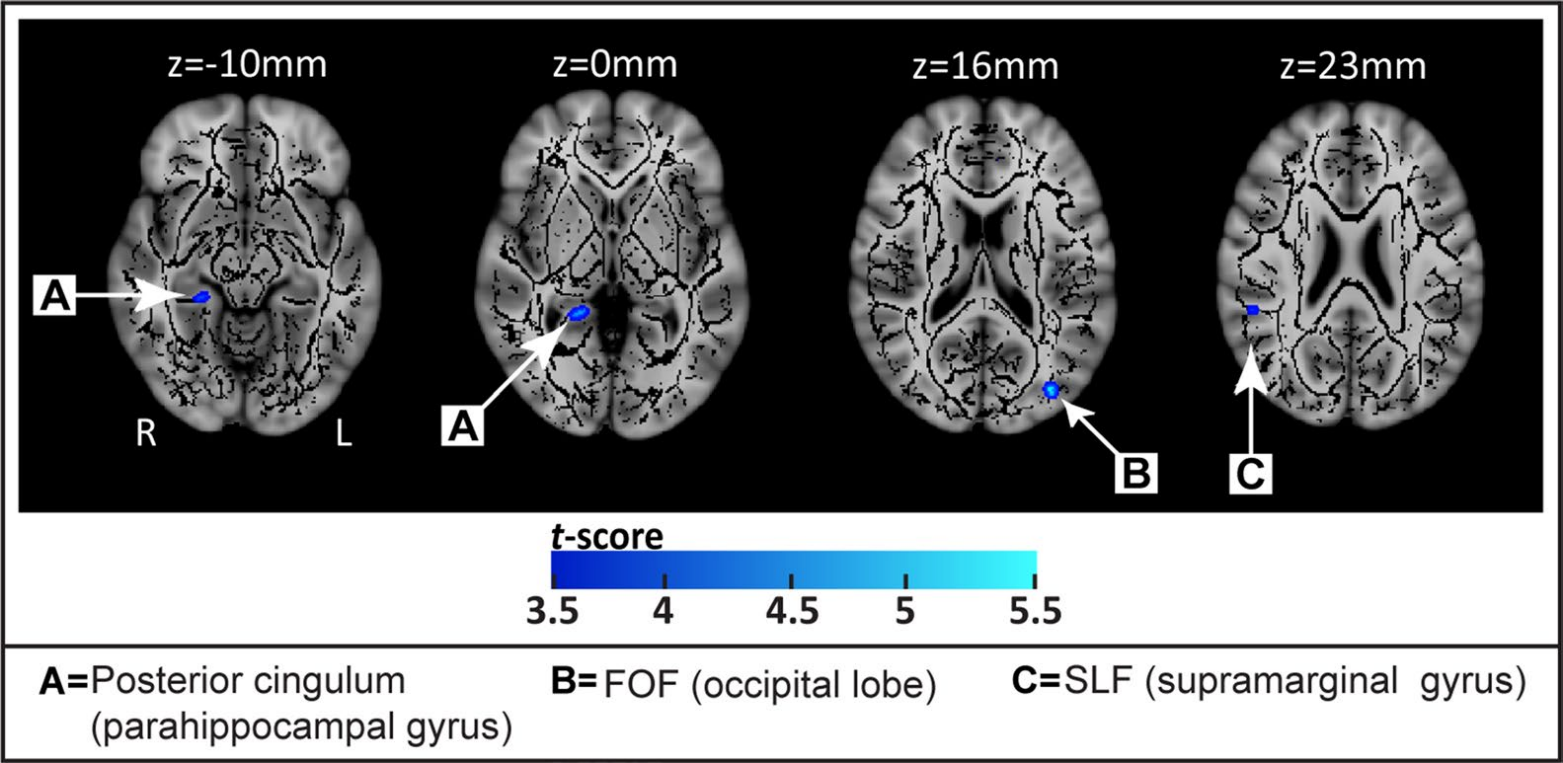
Regions with significantly reduced fractional anisotropy (FA) in rapists versus controls. The group mean FA skeleton (*black*) was overlaid on the MNI-152 T1 volume. The regions with significantly lower FA in rapists are *highlighted* with *colored voxels* (*blue* to *light blue*) corresponding to the t-scores. Significantly reduced FA was observed in the posterior cingulum (*A*), the inferior fronto-occipital fasciculus within the middle occipital gyrus (*B*), and the superior longitudinal fasciculus near the inferior parietal lobule (*C*)

Compared to the matched controls, sex offenders had smaller FA in several clusters. These include the left inferior fronto-occipital fasciculus of the occipital gyrus (p < 0.001), the right posterior cingulum of the parahippocampal gyrus (p < 0.001), and the right superior longitudinal fasciculus of the supramarginal gyrus (p < 0.001) (see Table 1; Fig. 1).

**Table 1:** Brain regions showing significant differences between rapist and control subjects, illustrating clusters in which rapists had reduced and increased white matter fractional anisotropy compared with control subjects

Cluster size (mm^3^)	MNI coordinates (mm)			Anatomical location		FA (Mean ± SD)		T score	*p* value	Cohen’s *d*
	x	y	z	White matter tract	Brain region	Control	Offender			
*Decreased FA in sex offenders versus controls*										
18	−35	−82	16	Inferior fronto-occipital fasciculus	Lateral occipital lobe, Middle occipital gyrus	0.42 ± 0.06	0.32 ± 0.03	5.55	0.0000	2.11
13	21	−43	0	Posterior cingulum	Parahippocampul gyrus	0.59 ± 0.05	0.50 ± 0.04	5.05	0.0001	1.99
10	52	−39	23	Superior longitudinal fasciculus	Inferior parietal lobule, or Supramarginal gyrus	0.54 ± 0.05	0.45 ± 0.05	4.26	0.0001	1.80
10	25	−35	−10	Posterior cingulum	Parahippocampul gyrus	0.60 ± 0.07	0.52 ± 0.05	3.39	0.0071	1.32
*Increased FA in sex offenders versus controls*										
29	−16	33	11	Genu		0.55 ± 0.05	0.63 ± 0.06	4.08	0.0001	1.45
17	12	8	1	Internal capsule (anterior thalamic radiation)	Caudate	0.62 ± 0.05	0.71 ± 0.04	4.45	0.0002	1.99
16	−25	−69	26		Lateral occipital lobe (superior division)	0.48 ± 0.05	0.56 ± 0.05	4.11	0.0002	1.60
16	18	−10	−3	Poterior limb of internal capsule	Globus pallidus	0.70 ± 0.05	0.76 ± 0.04	4.16	0.0003	1.33
14	24	−46	3	Posterior cingulate	Posterior cingulate	0.34 ± 0.09	0.46 ± 0.07	3.88	0.0028	1.49
14	−6	51	−18	Forceps minor	Medial frontal pole	0.23 ± 0.03	0.32 ± 0.07	4.06	0.0001	1.67
12	−7	−81	29	Forceps major	Cunerus	0.24 ± 0.04	0.33 ± 0.06	4.47	0.0002	1.77
11	42	−51	37	Superior longitudinal fasciculus	Supramarginal, or Angular gyrus	0.38 ± 0.09	0.51 ± 0.07	4.18	0.0006	1.61
10	−13	0	4	Internal capsule (anterior thalamic radiation)	Caudate	0.63 ± 0.05	0.71 ± 0.06	3.83	0.0025	1.45
10	−11	−5	−10	Posterior limb of internal capsule	Lower thalamus	0.69 ± 0.04	0.76 ± 0.05	3.37	0.0013	1.55

Covariate: age

**Fig. 2:**
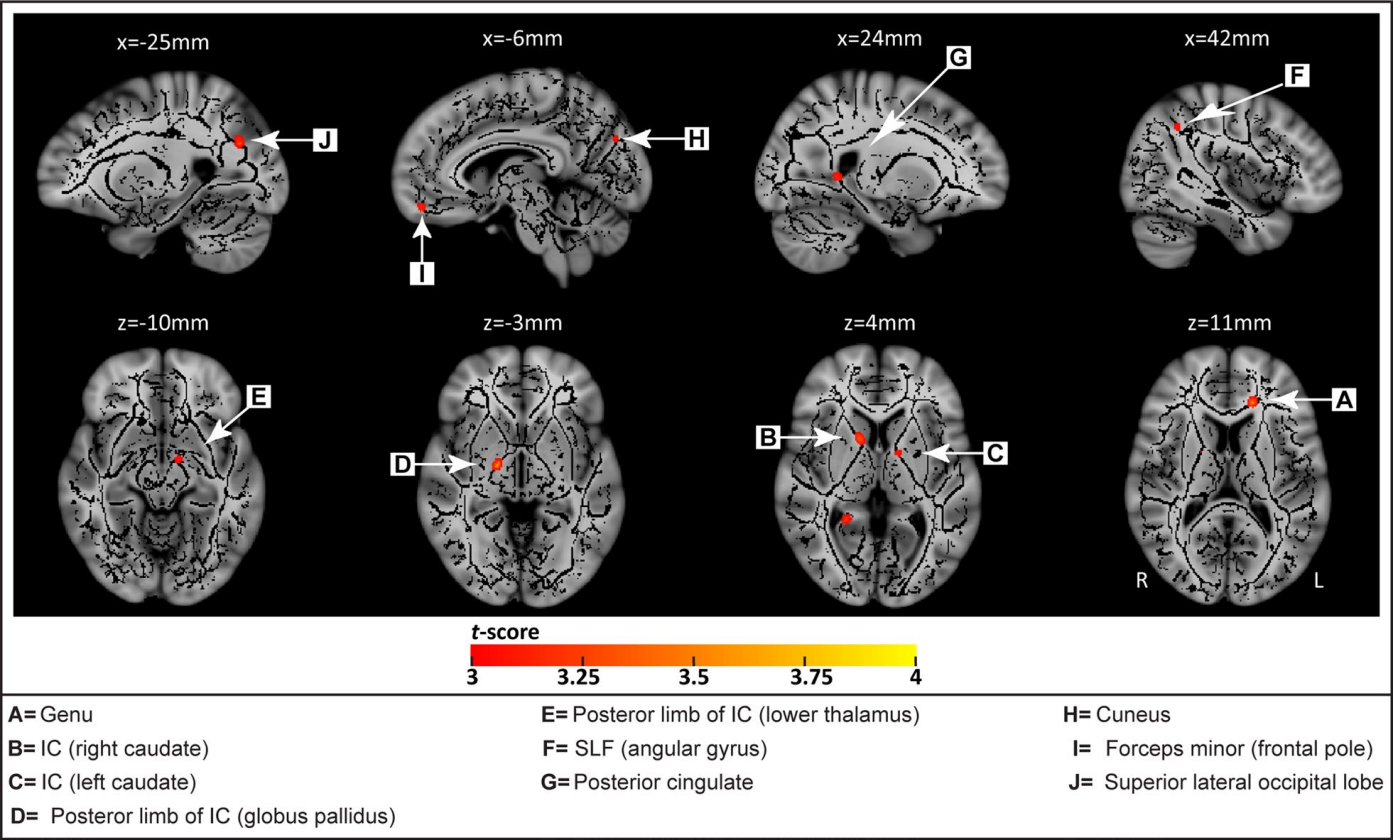
Regions with significantly increased fractional anisotropy (FA) in rapists versus controls. The group mean FA skeleton (*black*) was superimposed on the MNI-152 T1 image. The regions in color (*red* to *yellow*) represent the areas with significantly higher FA in rapists, including: white matter near the angular gyrus (*F*), the posterior cingulate (*G*), the medial frontal pole (*I*), and the internal capsule at the level of the thalamus (*E*), caudate (*B*, *C*), and globus pallidus (*D*)

Some regions showed greater FA in sex offenders compared to controls. These include clusters of the left superior longitudinal fasciculus adjacent to the angular gyrus (p < 0.001), the right posterior cingulate (p < 0.003), the forceps minor adjacent to the medial frontal pole (p < 0.001), and the right internal capsule at the level of the thalamus (p < 0.001), caudate (p < 0.001), and globus pallidus (p < 0.001) (see Table 1; Fig. 2).

Mean diffusivity (MD) was also analyzed but did not show significant differences in sexual offenders compared to the control group.

## Discussion

As predicted, results from this study revealed that male rapists show abnormalities in white matter integrity. Significant FA increases were found in: (a) white matter near the angular gyrus, posterior cingulate, and the medial frontal pole, and (b) the internal capsule at the level of the thalamus, caudate, and globus pallidus. Significantly reduced FA was observed in: (i) the posterior cingulum and (ii) the inferior fronto-occipital fasciculus within the middle occipital gyrus. To our knowledge, these constitute the first DTI findings on any sex offender group. These findings may be interpreted within the theoretical contexts of abnormalities in moral decision-making, sexual over-arousal, reward sensitivity, fear conditioning, and distorted social cognition in rapists.

Significant group differences were observed in white matter near the angular gyrus, the posterior cingulate, and the medial frontal pole. Previous studies using functional imaging have reported these regions to be involved in moral decision-making [18, 19]. These same regions have also been found to be dysfunctional in violent and psychopathic criminals [5]. It has also been argued that the rule-breaking behavior that is common in antisocial, violent, and psychopathic individuals, is in part due to impairments in these structures that underlie moral cognition and emotion [5]. Consequently, abnormality in white matter connectivity involving these regions could contribute to the antisocial, rule-breaking behavior found in rapists. While we found increased as opposed to decreased FA in rapists in these regions, it is conceivable that this increase could imply some dysfunction in the circuits associated with moral decision-making in this group. Deteriorated functions of these cortical regions could, for example, result in increased white matter connectivity, in an effort to compensate for this cortical dysfunction [20].

As indicated in Table 1, four of the 10 areas showing increased FA in rapists involved the internal capsule at the level of the thalamus, caudate, and globus pallidus. Activation of both the thalamus and caudate has been observed in normal men in response to viewing sexual material [21, 22]. Furthermore, at least one functional imaging study has shown that sex offenders registered over-activation of the thalamus, caudate, and globus pallidus during visual sexual stimulation [4]. Consequently, increased FA found in white matter tracts located near the thalamus, caudate, and globus pallidus may contribute to over-activation of brain areas related to sexual arousal. This over-activation could contribute to sexual over-arousal in offenders, which then acts as a trigger for actions leading to rape.

In this study, we observed increased FA in rapists in brain areas involved in the reward system, including the caudate, globus pallidus, and thalamus. Enlargement of the caudate has been associated with interpersonal and affective features of psychopathy, as well as impulsivity and stimulation seeking behavior [23]. Amongst sex offenders, rapists have been found to be more psychopathic than incest and pedophile offenders [24]. Heightened sensitivity to reward in rapists may, therefore, contribute to a heightened desire to pursue sexual rewards, and thus lead to rape in certain social circumstances.

Results from the current study showed that the sex offender group had increased FA in several regions of the internal capsule, when compared with the control group. Previous research on obsessive–compulsive disorder (OCD) has also indicated structural volumetric abnormalities of the internal capsule in patients with OCD [25], in addition to increased FA in this same region [26]. As a result, deep brain stimulation treatment of OCD has targeted the area of the internal capsule and the adjacent striatum. Similar to OCD patients, rapists have been hypothesized to have obsessive sexual thoughts [27]. Furthermore, OCD patients have been found to have increased FA in the internal capsule adjacent to the caudate, as well as reduced FA in the cingulum [28], a pattern also observed in rapists. It is conceivable therefore that abnormal FA in the internal capsule and cingulum could contribute to obsessive thinking on sexual themes, which may lead to a compulsion to rape.

When compared to controls, rapists also showed reduced FA in a number of white matter tracts in this study. First, they had reduced FA in the posterior cingulum. The posterior cingulum connects the cingulate and parahippocampal gyri to the septal cortex. As such, reduced FA may reflect an abnormality in septal functioning in sex offenders. Positioned alongside the hippocampus, the parahippocampal gyrus and the septum have been thought to play important roles in conditioning [29]. The reduced FA in the posterior cingulum shown in this study, that connects these structures, may suggest impairments in fear conditioning. Fear conditioning impairments as early as age 3 years have been found to predispose individuals to criminal behavior at age 23 [30]. Likewise, poor fear conditioning could predispose men to rape, because they do not experience anticipatory fear, and hence are less concerned about the consequences of their illegal behavior.

Abnormal posterior cingulum circuitry may result in reduced activation of the septum and a predisposition towards aggressive behavior. The same condition may be associated with increased activation of the anterior hypothalamus, and hence increased sexual arousal [31]. Recent research has documented abnormality in the septum in aggressive and psychopathic individuals, as indicated by cavum septum pellucidum [32], a neurological abnormality reflecting abnormal growth of limbic structures that include the septal nuclei, corpus callosum, hippocampus, amygdala, and other midline structures [33, 34]. In animals, the septum is critically involved in the regulation of aggression [35, 36]. Reduced FA in the posterior cingulum could therefore result in reduced activation of the septum and a predisposition towards aggressive behavior, as well as increased activation of the anterior hypothalamus and hence increased sexual arousal [31].

Finally, we found that rapists, when compared to controls, had decreased FA in the inferior fronto-occipital fasciculus within the middle occipital gyrus. This fiber tract has been found to be critically involved in the semantic processing and conceptualizing of visual stimuli [37, 38]. Distortions in social cognition have been found in rapists, which may serve as a basis and justification for their inappropriate sexual acts. An abnormality in the way rapists semantically process visual stimuli of a sexual nature in females (e.g. how they are dressed) could contribute to how they inappropriately mis-attribute the intentions of the women they meet.

Several limitations of the current study should be recognized. First, given the relatively modest sample size, replications involving larger sample sizes are recommended in order to confirm results from this study. Second, findings from adult male rapists cannot be generalized to other sexual offending groups, including pedophiles and homosexual rapists. Third, while results indicated a number of significant abnormalities in white matter integrity in rapists, our study cannot delineate the cause of these abnormalities. Fourth, the hypothesis that increased FA in the internal capsule in rapists may contribute to obsessive sexual thinking must be regarded as preliminary and requiring further substantiation.

Additionally, like any analytical method, there is potential for improvement in future investigations, based both on the findings from the study here, and refinement using data collected employing additional or alternative methods. For the TBSS analysis, an approach using registration to either the FMRIB58 template or to the most representative subject of the group may be beneficial. Alternatively, replacing the TBSS registration step with a tensor-based, group-wise registration, e.g. using DTI-TK, may be an alternative, particularly given that reliability for low-FA structures like the fornix or the external capsule can be quite low [39]. Additionally, a combination of approaches using fMRI (both active and resting) and VBM may provide information for selection of areas for specific-tract tractography, and so extend the range of the current findings. It is important to remember, however, that the TBSS analysis employed here required no assumptions to be made regarding regions that may be affected, whereas a more specific hypothesis would be required for tractography analysis.

Despite these limitations, however, this study provides the first demonstration of abnormalities in the structural integrity of white matter in the brains of rapists. Inclusion criteria that restricted participants to adult males perpetrating their offense on a female stranger resulted in a relatively homogenous group in contrast to some other neurobiological studies that examined more heterogeneous groups of sex offenders [e.g. 40]. Results from this study encourage future research investigating white matter integrity among sexual offenders.

## Conclusion

In this study, significant differences in FA between male rapists and the control group were seen in a number of areas including white matter near the angular gyrus, posterior cingulate, the medial frontal pole, the internal capsule at the level of the thalamus, caudate, and globus pallidus, the posterior cingulum, and the inferior fronto-occipital fasciculus within the middle occipital gyrus. A subset of these areas (the angular gyrus, posterior cingulate, and the medial frontal pole) have previously been found to be dysfunctional in violent and psychopathic criminals, consistent with the functional relevance of these areas to criminal behavior. Differences in the remaining areas may relate to, for example, a tendency for sexual over-arousal, heightened sensitivity to reward, obsessive thinking on sexual themes, and poor fear conditioning, all of which may potentially contribute to the nature of the criminal behavior perpetrated by these individuals. Future work to investigate potential causes of these differences, as well as contrasting rapists with different criminal populations, would be beneficial.

## Abbreviations

FA: fractional anisotropy
DTI: diffusion tensor imaging
MRI: magnetic resonance imaging
T1W: T1-weighted
FLAIR-FSPGR: fast spoiled gradient recalled echo
TR: repetition time
TE: echo time
TI: inversion time
EPI: echo-planar imaging
BET: brain extraction tool
FSL: functional magnetic resonance imaging of the brain software library
TBSS: tract-based spatial statistics
OCD: obsessive–compulsive disorder
